# Systemic Lupus Erythematosus Presenting as Walking Corpse Syndrome

**DOI:** 10.7759/cureus.39840

**Published:** 2023-06-01

**Authors:** Barbara Luma, Kedar Challakere, Joshua Mandap, Sree Datla

**Affiliations:** 1 Behavioral Health, Arrowhead Regional Medical Center, Colton, USA; 2 Psychiatry and Behavioral Sciences, California University of Science and Medicine, Colton, USA; 3 Psychiatry, California University of Science and Medicine, Colton, USA

**Keywords:** psychosis, walking corpse syndrome, lupus cerebritis, systemic lupus erythematosus, cotard syndrome

## Abstract

Cotard syndrome, also known as “Walking Corpse Syndrome”, is a clinical entity characterized by fixed delusions that one is dead or dying. This is a neuropsychiatric manifestation of brain pathology affecting the non-dominant frontotemporal and parietal lobes, particularly the fusiform gyrus. Prior literature has indicated that the etiology of Cotard syndrome may include structural changes related to brain injury, tumors, and temporal lobe epilepsy. We now present a case in which Cotard syndrome is related to systemic lupus erythematosus (SLE). Neuropsychiatric symptoms are atypical manifestations of SLE. Delusions, hallucinations, and other psychotic symptoms can result as a consequence of the disease itself or from corticosteroid treatment. A diagnosis of SLE-induced psychosis can be elusive; however, conducting a thorough workup is crucial as untreated psychosis secondary to lupus cerebritis can worsen without intervention. We present a clinical unique case of SLE cerebritis, diagnostic challenge, and management.

## Introduction

Systemic lupus erythematosus (SLE) is an autoimmune inflammatory condition with widespread, variable clinical manifestations across multiple organ systems. SLE primarily affects women of childbearing age, particularly women of Hispanic, African-American, and Asian descent [1]. While often unrecognized, clinically significant neuropsychiatric symptoms may be present in up to two-thirds of patients [2], with psychosis present in approximately 12% of cases [3]. In 1999, The American College of Rheumatology established the following standardized guidelines to assess psychosis in the context of SLE: 1) Delusions or hallucinations without insight; 2) resulting in clinical distress or impairment in social, occupational, or other relevant areas of functioning; 3) disturbance should not occur exclusively during delirium; and 4) not better accounted for by another mental disorder. Based on these criteria, we present the case of a 48-year-old Hispanic woman with SLE-induced psychosis.

## Case presentation

A 48-year-old Hispanic female was brought in by her family in May 2022 for altered mental status of approximately one-week duration preceded by lower extremity edema and a five-month history of decreased food intake with weight loss. Upon initial assessment, the patient was alert but only able to state her name. She was unable to verbalize the year, place, or situation. She repeatedly stated, “I live on the planet in the sky.” She was also overheard by her family stating that her daughter and husband were dead when, in fact, her daughter was beside her. 

Per family, her premorbid social functioning included a high level of social functioning including the ability to respond and interact appropriately with family members and others. Of interest, the patient had been previously hospitalized in February 2022 at an outside hospital for lower extremity edema with her body “tightening up.” No formal diagnosis was established at the time of this February admission. Per chart review, during that hospitalization, the patient was placed on a five-day course of prednisone 20 mg per day.

Apart from the February hospitalization, the history obtained from the family revealed that the patient had no pertinent past medical, surgical history, or substance use. Family history was non-contributory for psychiatric or autoimmune disorders.

Physical examination was remarkable for GCS of 14 with patient alert and oriented to name only, central hair loss, tachycardia of 114 bpm, swan neck deformity in the left second and third digits, and 4+ bilateral pitting edema of her lower extremities. She reported of tiredness and general weakness. Laboratory values were remarkable for hemoglobin of 6.9 resulting in the administration of 1 unit of packed RBCs, BNP of 3,726, creatinine of 1.82, BUN of 45, alkaline phosphatase of 162, AST of 68, albumin of 3.0, lactate of 2.80, and TSH of 7.18. All lab findings are summarized in Table 1, and pertinent findings from the hospital course are shown in Table 2. CT scan non-contrast of the head and brain was unremarkable. In the emergency department, the patient was administered Ativan 1 mg IM to improve agitation, diagnosed with unspecified altered mental status, and admitted to the internal medicine service. 

**Table 1:** Pertinent lab values during admission BNP: B-type natriuretic peptide; BUN: blood urea nitrogen; ALP: alkaline phosphatase; AST: aspartate aminotransferase; TSH: thyroid-stimulating hormone

Lab	Hgb (g/dL)	BNP (pg/ml)	Creatinine (mg/dL)	BUN (mg/dL)	ALP (units/L)	AST (units/L)	Albumin (g/dL)	Lactate (mmol/L)	TSH (µIU/mL)	B12 (pg/mL)
Patient Value	6.9	3,726	1.82	45	162	68	3.0	2.8	7.18	>2,000
Normal Range	12.1-15.1	<100	0.6-1.12	5-20	30-120	0-35	3.5-5.0	<2	0.35-5	160-950

**Table 2:** Pertinent lab values during the hospital course TSH: Thyroid-stimulating hormone; ESR: erythrocyte sedimentation rate

Lab	TSH (µIU/mL)	ESR (mm/hr)	ANA titer	anti-dsDNA Ab (IU/mL)	Anti-smith Ab (IU/mL)	C3 (mg/dL)	C4 (mg/dL)	PS lgM Ab (U/mL)	LP WBC count	LP glucose (mg/dL)	LP RBC count	24 hrs Urine protein (mg)	Urine Creatinine (mg)	Albumin/Cr ratio
Patient Value	9.73	140	1:320	807	8	23	5	61	0	40	9	1288	360	420.4
Normal Range	0.35-5	0-29	<=1:40	<30	0-7	80-178	12-42	0-25	0-5	2/3 of Serum	<1	<150	604-1,689	<30

Initial differential diagnoses were non-specific, with differential diagnoses encompassing sepsis versus dehydration versus less likely vitamin B12 deficiency. The patient became febrile on day 7 of hospitalization, and her chest X-ray revealed left lower lobe pneumonia. Vancomycin 750 mg every 24 hours and cefepime 2g were started IV every 12 hours. Additional diagnostic evaluations revealed negative syphilis and HIV screens, absence of growth in blood cultures at 48 hours, and B12 level greater than 2,000, thus ruling out suspected B12 deficiency. Due to lack of growth in blood cultures, antibiotics were discontinued. 

The patient continued to state that both she and her family were dead. She refused to eat and thought her meals were being poisoned; at times, she stated that she was already dead and had no need to eat. With consent from both family and the patient, a nasogastric tube for feeding was placed. The patient subsequently began to report that she was being poisoned by the nutrients delivered via the tube.

The psychiatric consultant recommended initiation of risperidone 0.5 mg PO BID which resulted in no improvement of her neuropsychiatric symptoms. The neurological consultant recommended an MRI of the brain which was unremarkable. MRA of the brain, however, revealed possible proximal arterial thrombosis versus arteriovenous malformation. Given multiple organ system involvements and lack of evidence for primary psychotic, metabolic, or infectious causes, and her physical exam findings, autoimmune etiology was considered; laboratory testing for ESR, ANA, anti-dsDNA antibody, anti-Smith antibody, C3 & C4 levels, and SSA antibody was ordered along with CSF studies. 

Continued laboratory evaluations revealed increasing TSH with a value of 9.73, elevated ESR at 140, ANA titer at 1:320, anti-dsDNA antibody at 807, elevated anti-smith antibody levels with a value of 8 IU/ml, low C3 of 23 mg/dL, low C4 of 5 mg/dL, positive phosphatidylserine IgM of 61U/ml, and positive SSA antibody. Lumbar puncture revealed a lack of white blood cells, a glucose level of 40, and a red blood cell count of 9. MRI of the brain with gadolinium contrast was ordered, along with oligoclonal bands and anti-ribosomal P antibodies in CSF, for evidence of CNS involvement. This MRI revealed no acute cerebral infarction, intracranial mass, or hemorrhage.

Based on these criteria, the patient met the criteria for a diagnosis of SLE based on the 2019 guidelines of the American College of Rheumatology [4]. She also met clinical criteria in the neuropsychiatric domain (delirium, psychosis), mucocutaneous domain (non-scarring alopecia), and renal domain (proteinuria >0.5 g/24 hours). The systemic lupus erythematosus disease activity index (SLEDAI) score of 26 was consistent with severe disease activity. 

The rheumatological consultant concurred with a diagnosis of lupus cerebritis based on the above findings and recommended initiation of Solu-Medrol 50 mg daily intravenous infusion. 

**Figure 1:**
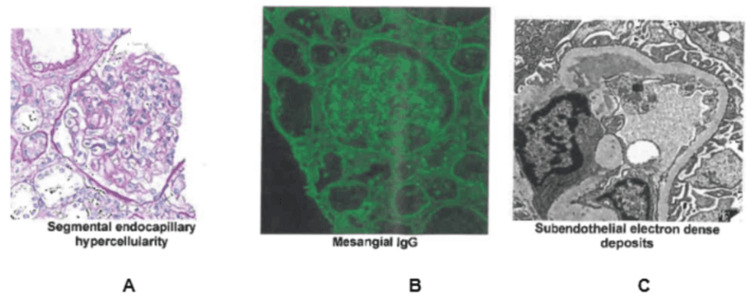
Renal biopsy results. Findings consistent with focal active lupus nephritis, ISN/RPS Class III A. (A) Segmental endocapillary hypercellularity. (B) Mesangial IgG. (C) Subendothelial electron dense deposit

Due to abnormal renal function, including proteinuria of 1.3g/24 hr, 24 hrs urine protein 1288, Cr 360, and albumin: creatinine ratio of 420.4, nephrology was consulted and recommended renal biopsy in June 2022. The biopsy showed class 3 lupus nephritis as shown in Figure 1, and the patient was subsequently started on cyclophosphamide 500 mg IM once per rheumatology recommendation. Her renal biopsy showed focal active lupus nephritis, with 5% global sclerosis. The patient continues to be hospitalized at the time of case report preparation secondary to necrotizing fasciitis with her last urine protein trending downwards to 26 mg/dl in August 2022, indicating possible improvement after cyclophosphamide was started. 

## Discussion

This patient initially presented to the emergency department due to a week of altered mental status. Despite a history of good premorbid cognitive and interpersonal functioning, at admission, she was only oriented to herself. Alternative diagnoses such as organic psychosis, substance-induced mental status changes, and medication-induced psychoses were considered. Based on the negative workup of other causes such as sepsis and dehydration, along with findings on rheumatological laboratory values and neuroimaging, a diagnosis of SLE-induced cerebritis with psychosis was established. The basis for this diagnosis included elevated ANA, elevated anti-dsDNA antibody of 807, elevated anti-Smith antibody greater than 8, low C3 of 23, low C4 of 5, positive phosphatidylserine IgM of 61, and positive SSA antibody, along with MRI findings. 

The presence of nihilistic delusions such as the patient herself and family members being deceased along with beliefs that eating was pointless since she was already dead revealed poor insight, thus meeting ACR criterion 1 for psychosis in the presence of SLE [5-7]. This patient’s decline in social functioning met ACR criterion 2. Consistency of patient’s delusions unrelated to fluctuations in level of consciousness met ACR criterion 3. The laboratory and neuroimaging studies ruled out causes other than SLE (criterion 4).

A diagnosis of SLE cerebritis with psychotic manifestations requires high clinical suspicion and has presentations that can include any of the following: Confusion, cognitive dysfunction, mood changes, lethargy, seizures, or coma [7,8]. Previous case reports of SLE psychosis corroborate this, including a temporal relationship between underlying disease and the onset of symptoms [9]. This patient’s SLE presentation was especially notable for several reasons. Neuropsychiatric manifestations as a result of SLE are rare events that occur only in around 2% of patients [5,10]. Lupus cerebritis is less common for a woman of 44 years of age to develop lupus cerebritis than with the pediatric and young adult populations, who are at greater stochastic risk for lupus cerebritis [11,12]. In addition, MRI of the brain can be normal in up to 42% of cases despite having signs and symptoms of active disease [2,12].

The standard treatment for neuropsychiatric systemic lupus erythematosus (NPSLE) is intravenous methylprednisolone (IVMP) given at a dose of 1g daily for 3-5 days [10]. This patient was started on an immunosuppressive regimen with cyclophosphamide, hydroxychloroquine, mycophenolate, and prednisone with her most recent urine protein trending downwards, indicating possible improvement regarding her lupus cerebritis and nephritis.

## Conclusions

SLE is a chronic immunologic disease with multi-organ system involvement. It can manifest with neuropsychiatric presentations, deemed NPSLE, with one rare possibility being psychosis. In our case above, we presented the clinical case of a middle-aged woman with hallucinations, swan neck deformities in the left second and third digits, bilateral lower extremity pitting edema, and facial skin tightening with telangiectasias. Combined with lab results of elevated ANA, elevated anti-dsDNA Ab, elevated anti-Smith Ab, low C3/C4, and positive phosphatidylserine IgM, along with neuroimaging, a diagnosis of lupus cerebritis with psychotic features was identified. Prompt, proper diagnosis of neuropsychiatric manifestations of SLE has important implications including the fact that patients with SLE have higher rates of major depressive disorder with or without psychotic features and carry an increased risk of suicide. We would like to present the investigational process and treatment as delineated above as a potential guideline in the diagnosis of this rare but serious condition.
